# A mouse model of systemic lupus erythematosus responds better to soluble TACI than to soluble BAFFR, correlating with depletion of plasma cells

**DOI:** 10.1002/eji.201746934

**Published:** 2017-04-24

**Authors:** Philipp Haselmayer, Michele Vigolo, Josquin Nys, Pascal Schneider, Henry Hess

**Affiliations:** ^1^ Department of Immunopharmacology Immunology Translational Innovation Platform Merck KGaA, Darmstadt Germany; ^2^ Department of Biochemistry University of Lausanne Epalinges Switzerland

**Keywords:** APRIL, BAFF/BLyS, Lupus, Plasma cell, SLE

## Abstract

The TNF family cytokines B‐cell activating factor (BAFF) and a proliferation‐inducing ligand (APRIL) support plasma cell survival. It is known that inhibitors of BAFF only (BAFFR‐Fc) or BAFF and APRIL (TACI‐Fc) administered early enough in an NZB/NZW F1 mouse model of systemic lupus erythematosus (SLE) ameliorate clinical outcomes, pointing to a pathogenic role of BAFF. In the present study, TACI‐Fc administrated at a later stage of disease, after onset of autoimmunity, decreased the number of bone marrow plasma cells and slowed down further formation of autoantibodies. TACI‐Fc prevented renal damage during a 12‐week treatment period regardless of autoantibody levels, while BAFFR‐Fc did not despite a similar BAFF‐blocking activity in vivo. TACI‐Fc also decreased established plasma cells in a T‐dependent hapten/carrier immunization system better than single inhibitors of BAFF or APRIL, and sometimes better than combined single inhibitors with at least equivalent BAFF and APRIL inhibitory activities. These results indicate that TACI‐Fc can prevent symptoms of renal damage in a mouse model of SLE when BAFFR‐Fc cannot, and point to a plasticity of plasma cells for survival factors. Targeting plasma cells with TACI‐Fc might be beneficial to prevent autoantibody‐mediated damages in SLE.

## Introduction

The homotrimeric TNF family ligands BAFF (B cell‐activating factor of the TNF family, also known as B Lymphocyte stimulator) 1, 2 and APRIL (A proliferation‐inducing ligand) 3 are important differentiation and survival cytokines for B cells. BAFF and APRIL can also form heterotrimers whose biological relevance remains unknown 4, 5, 6. BAFF engages three specific receptors, BCMA (B cell maturation antigen) 7, 8, TACI (transmembrane activator and CAML interactor) 9 and BAFFR (BAFF receptor) 10, while APRIL binds to BCMA, TACI and, via another portion of its structure, to sulfated glycosidic chains of proteoglycans 11, 12. BAFF‐deficient mice have reduced numbers of peripheral B cells, showing that BAFF is required for mature B cell survival in vivo 13, 14. The phenotype of APRIL‐deficient mice is milder, with impaired class switch recombination to IgA 15. It is BAFFR that mediates BAFF survival signals in transitional and mature B cells 10, 16. TACI is required for T‐independent antibody responses and to negatively regulate the B‐cell pool 17, 18. BCMA contributes to the maintenance of terminally differentiated plasma cells (PCs) 19. APRIL, together with IL‐6 and stromal cell‐derived factors can be used to generate and indefinitely support human long‐lived PC in vitro 20.

In the population of patients suffering from certain autoimmune diseases, elevated levels of soluble BAFF and/or APRIL can be detected 21, 22, 23. Mice genetically modified to over‐express BAFF develop symptoms of a systemic lupus erythematosus (SLE)‐like autoimmunity 7, 24. Excess BAFF may favor autoimmunity via partial subversion of B‐cell self‐tolerance at the level of naïve B cells 25. At the level of antigen‐experienced mature B cells, a model of competitive elimination for chronically stimulated B cells, which require BAFF for survival, has been proposed 26. BAFF antagonism has proven efficacious in a number of different animal models for human SLE 27, 28, 29, 30, 31, while selective neutralization of APRIL in a mouse model of SLE only modestly delayed disease apparition 32. In a particular congenic inbred mouse strain, which serves as a model for human SLE, BAFF, APRIL, and their receptors have been genetically deleted 33, 34, 35, 36, 37. Deletion of APRIL, BAFFR, BCMA, or TACI did not ameliorate SLE. Deletion of BAFF delayed apparition of autoantibodies and deposition of immune complexes, and attenuated disease severity 35, as did deletion of BAFFR plus either BCMA or TACI 37. Deletion of both BAFF and APRIL decreased the bone marrow PC compartment and strongly reduced IgG anti‐chromatin and anti‐dsDNA antibody levels, an effect not observed by deletion of BAFF or APRIL separately 34, 35. These data show that maximal suppression of autoimmunity is achieved by dual BAFF and APRIL inhibition, even though this did not further improved health of mice compared to BAFF deletion alone in the experimental window examined and raised the concern of side effects caused by excessive depression of the humoral response.

In the present study, we compared pharmacological inhibition of BAFF (using mouse BAFFR‐Fc, mBAFFR‐Fc), or of BAFF and APRIL (using mouse TACI‐Fc, mTACI‐Fc) in SLE‐prone mice with declared autoimmunity and found that TACI‐Fc, but not BAFFR‐Fc, prevented renal pathology even in the presence of autoantibodies. We also report that in immunized, non‐autoimmune mice, 2 weeks’ treatment with TACI‐Fc, but not with BAFFR‐Fc, decreased both recently‐generated and long‐lived PCs in the bone marrow, but that the latter were not depleted by the combined action of single BAFF and APRIL inhibitors. We conclude that, at least under specific circumstances, TACI‐Fc does more than single BAFF inhibition to reduce PCs and to decrease SLE symptoms. Our results also suggest that PC responses to survival factors can change as they age.

## Results

### In vitro characterization of a function‐blocking anti‐mouse APRIL antibody

Because BCMA participates to survival and longevity of PCs in the bone marrow 19 and displays a higher affinity for APRIL than for BAFF 38, APRIL is likely to transmit survival signal to PCs. We characterized an antibody suitable for pharmacological inhibition of mouse APRIL. This reagent, Apry‐1‐1, is a human single chain monoclonal antibody dimerized by fusion to the Fc portion of mouse IgG2b. Apry‐1‐1 specifically recognized and inhibited mouse APRIL in a variety of settings in vitro and compared favorably with hTACI‐Fc in terms of mouse APRIL inhibition (Supporting Information Fig. 1).

### TACI‐Fc, but not BAFFR‐Fc, arrests disease progression in NZB/NZW F1 mice

**Figure 1:**
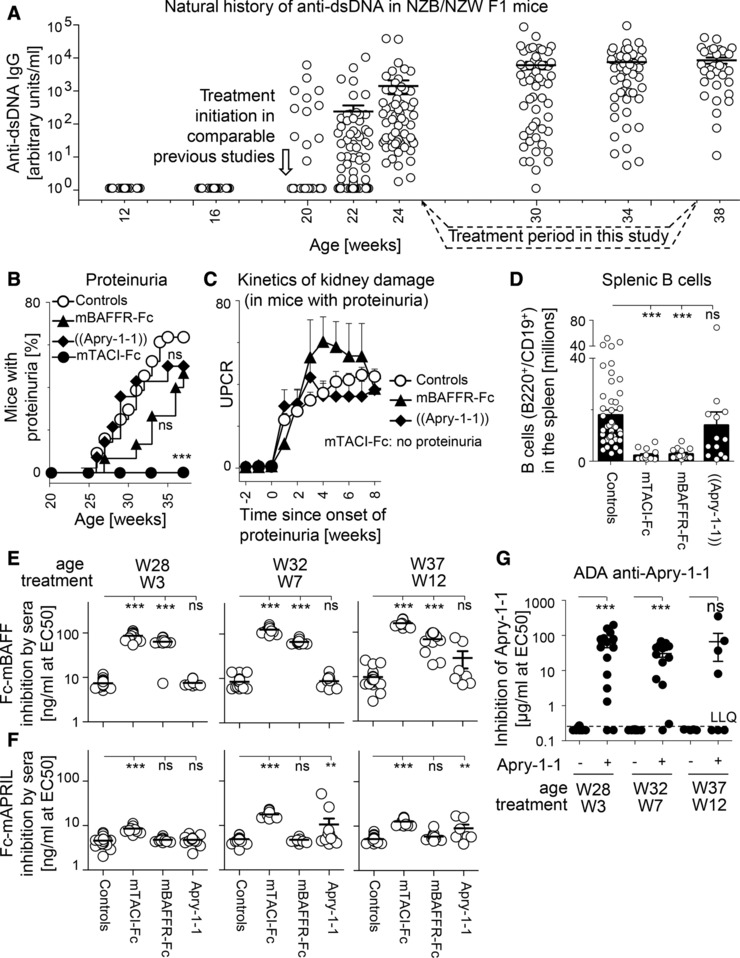
mTACI‐Fc, not mBAFFR‐Fc, prevents proteinuria in NZB/NZW F1 mice with declared autoimmunity, while anti‐APRIL Apry‐1‐1 is neutralized by an anti‐drug antibody response. (A). Longitudinal assessment of anti‐dsDNA antibody levels in NZB/NZW F1 mice. Sera were prepared from cohorts of NZB/NZW F1 mice at the indicated ages. Relative titers of anti‐dsDNA IgG were determined by ELISA in triplicate titrations. Pooled data from controls of 4 experiments. Number of sera analyzed at weeks 12/16/20/22/24/30/34/38 were 70/65/25/70/60/55/49/31. (B). NZB/NZW F1 mice were treated for 12 weeks, three times a week, with mTACI‐Fc, mBAFFR‐Fc, anti‐mAPRIL Apry‐1‐1 or appropriate controls starting at 25 weeks of age (*n* = 15 for mTACI‐Fc and mBAFFR‐Fc, *n* = 14 for Apry‐1‐1, *n* = 44 for pooled mFc, mIgG and untreated controls), when the majority of mice were positive for anti‐dsDNA and negative for proteinuria. Kaplan‐Meier plot depicting the fraction of mice over time that developed proteinuria (defined as UPCR ≥ 3). (C). Kinetics of urinary protein to creatinine ratio (UPCR) increase, where week 1 is defined as the first week when a given mouse had a UPCR ≥3. Only the subset of mice shown in panel 1B that developed proteinuria is analyzed (at week 1, *n* = 28 for controls, *n* = 7 for BAFFR‐Fc, *n* = 6 for Apry‐1‐1). Mice treated with mTACI‐Fc do not appear on this graph because they did not develop proteinuria. UPCR was measured once per mouse and time point. (D). Absolute B cell numbers (CD19^+^ and B220^+^) found in the spleen of NZB/NZW F1 mice as determined by FACS analysis on the day of sacrifice at 12 weeks of treatment (n for controls/TACI‐Fc/BAFFR‐Fc/Apry‐1‐1: 26/15/11/4) or before 12 weeks of treatment (n for controls/TACI‐Fc/BAFFR‐Fc/Apry‐1‐1: 18/0/4/9). (E). Amounts of mBAFF‐neutralizing activities were measured at weeks 3, 7 and 12 of the indicated treatments using a cell‐based reporter assay (BCMA:Fas reporter cells). Each point represents the EC50 of a titration of recombinant Fc‐mBAFF performed on BCMA:Fas reporter cells in the presence of serum diluted 1/300. Number of sera analyzed for controls/TACI‐Fc/BAFFR‐Fc/Apry‐1‐1 at weeks 3, 7 and 12 were 45/15/15/15, 40/15/15/13 and 25/15/14/7, respectively. Sera of mice sacrificed before 3, 7, and 12 weeks of treatment were respectively assigned to groups 3, 7, and 12 weeks. Each value was obtained from the EC50 of a titration performed once. (F). Same as panel E, but for the measure of Fc‐mAPRIL‐neutralizing activity. (G). Quantification of anti‐drug antibody (ADA) response directed against Apry‐1‐1 in sera of mice treated for 3, 7, or 12 weeks with Apry‐1‐1 or in untreated controls. For that purpose, BCMA:Fas reporter cells were exposed to a fixed lethal concentration of Fc‐mAPRIL, but rescued in the presence of titrated concentrations of pure Apry‐1‐1. The anti‐Apry‐1‐1 ADA response was measured as the capacity of sera diluted 1/300 to prevent rescue of reporter cells by pure Apry‐1‐1. Number of sera analyzed for untreated controls/Apry‐1‐1 at weeks 3, 7 and 12 were 15/15, 13/13, and 4/7, respectively. Each value was obtained from the EC50 of a titration performed once. Panels A and D‐G show mean of each group ± SEM, with symbols representing individual mice. Panel C shows mean ± SD. The experiment analyzed in panels 1B ‐ 1G was performed once. Analyses were performed once, except those of panels E‐G that were performed twice with similar results in two independent sets of measurements of the same set of sera. Statistical analysis was performed with Mantel‐Cox test (B), one‐way ANOVA followed by Bonferroni comparing controls to each treatment (D‐F), and unpaired *t*‐test comparing untreated to treated (G). ns: nonsignificant; *p < 0.05; **p < 0.01; ***p < 0.001.

The NZB/NZW F1 mouse model of SLE was used to compare pharmacological inhibitors and assess the contributions of BAFF and APRIL to disease progression. In the absence of treatment, NZB/NZW F1 mice started to produce anti‐dsDNA IgGs at 20‐ to 22‐week‐old, and most of them were positive at week 24 (Fig. 1A). At that stage, mice were still negative for proteinuria (Fig. 1B) and corresponded in terms of clinical manifestations to patients with low disease activity. Mice were separated in groups with comparable autoantibody distributions and were treated from week 25 to week 37 with inhibitors of BAFF and/or APRIL. The different control groups were pooled for the analysis because we noticed no difference between them. Mice treated with mTACI‐Fc remained negative for proteinuria at all time points, in contrast to controls in which the percentage of affected mice steadily increased up to week 37 (Fig. 1B). Mice treated with Apry‐1‐1 were as affected as controls, and mice receiving mBAFFR‐Fc only showed a non‐significant trend to delayed onset of proteinuria (Fig. 1B). In all unprotected groups including the one treated with mBAFFR‐Fc, once proteinuria became positive, UPCR increased to high values over just 1–2 weeks (Fig. 1C).

### TACI‐Fc and BAFFR‐Fc inhibit BAFF similarly, but Apry‐1‐1 is inactivated in NZB/NZW F1 mice

Anti‐BAFF activity in vivo can be visualized by depletion of mature splenic B cells within 2 weeks 39. In mice treated with mTACI‐Fc or mBAFFR‐Fc, splenic B cells were depleted (Fig. 1D, Supporting Information Fig. 2). Anti‐BAFF and anti‐APRIL inhibitory activities were also measured directly in sera at three different time points of treatment using a cell‐based assay. Briefly, BCMA:Fas reporter cells exposed to recombinant BAFF or APRIL die by activation of the surrogate Fas apoptotic pathway. Active inhibitors in sera are monitored for their capacity to protect reporter cells from BAFF‐ or APRIL‐mediated death. Anti‐mBAFF activities were comparable in mTACI‐Fc‐ and mBAFFR‐Fc‐treated mice, slightly higher in the mTACI‐Fc group, but in any case present in excess (Fig. 1E). mTACI‐Fc had a low but significant mAPRIL inhibitory action. Unexpectedly, Apry‐1‐1 treatment generated no anti‐APRIL activity in sera of most treated mice (Fig. 1F), even though Apry‐1‐1 was superior to mTACI‐Fc in vitro (Supporting Information Fig. 1H). Instead, sera of Apry‐1‐1‐treated animals could inhibit exogenously added Apry‐1‐1 up to high levels in the reporter cell assay (Fig 1G). This strongly suggests that autoimmune NZB/NZW F1 mice mounted a rapid and efficient anti‐drug antibody response against Apry‐1‐1, thus invalidating this arm of the study. Results obtained with Apry‐1‐1 treatment are therefore shown with the indication of Apry‐1‐1 in double brackets to indicate that the active agent had been neutralized. In contrast, excess neutralizing activity against mTACI‐Fc or mBAFFR‐Fc was not detected, as demonstrated by the positive anti‐BAFF activity present in these samples (Fig. 1E).

### TACI‐Fc diminishes bone marrow PC numbers and stabilizes autoantibody secretion in NZB/NZW F1 mice

**Figure 2:**
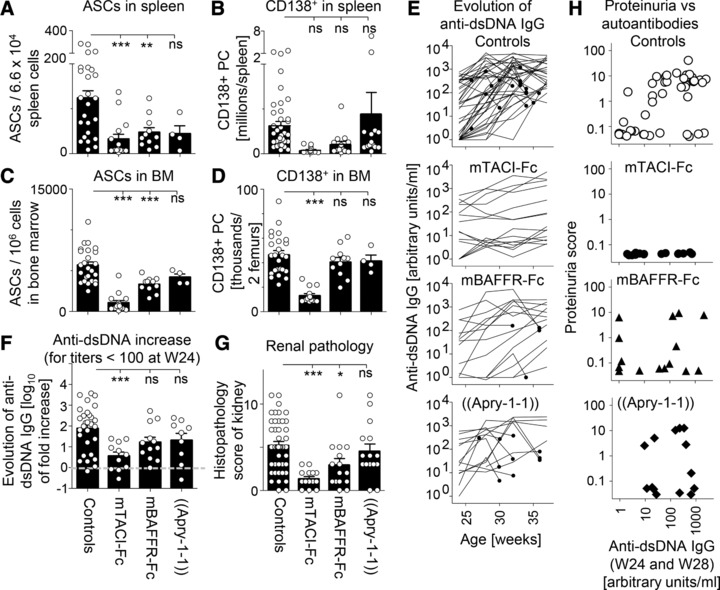
mTACI‐Fc is more effective than mBAFFR‐Fc in diminishing the bone marrow PC compartment and in preventing autoantibody increase and renal immunopathology in NZB/NZW F1 mice. (A). Antibody secreting cells (ASC) retrieved from spleens of 37‐week‐old NZB/NZW F1 mice subjected to the indicated treatments, as determined by ELISPOT. n for controls/TACI‐Fc/BAFFR‐Fc/Apry‐1‐1 were 25/15/11/4. Values were obtained from titrations performed in triplicates. (B). Numbers of PCs (CD138^+^) derived from spleens of NZB/NZW F1 mice. Absolute cell numbers were determined by FACS from specimens taken at termination of the experiment and included analyses of animals that had been sacrificed at earlier times throughout the duration of the study. n for controls/TACI‐Fc/BAFFR‐Fc/Apry‐1‐1 were 44/15/15/14. (C). Same as panel A, but for the bone marrow of 37‐week‐old mice. (D). Same as panel B, but for absolute numbers of CD138^+^ cells in the bone marrow of two femurs and for 37‐week‐old mice only. n for controls/TACI‐Fc/BAFFR‐Fc/Apry‐1‐1 were 25/15/11/4. (E). Evolution of anti‐dsDNA IgG levels in groups of control and treated mice. Mice that had to be sacrificed before week 37 are indicated with black circles. n for controls/TACI‐Fc/BAFFR‐Fc/Apry‐1‐1 were 44/15/15/14. (F). Log_10_ of fold change of anti‐dsDNA levels between week 24 and week 37. Only NZB/NZW F1 mice with less than 100 arbitrary units of anti‐dsDNA antibody at week 24 were included in this analysis. n for controls/TACI‐Fc/BAFFR‐Fc/Apry‐1‐1 were 28/10/12/8. (G). Renal histology scores for the different treatment groups at sacrifice (week 37 or earlier). n for controls/TACI‐Fc/BAFFR‐Fc/Apry‐1‐1 were 45/15/15/15. (H). Relationship between anti‐dsDNA titers at early time points (average of titers measured at weeks 24 and 28) and a proteinuria score taking into account timing of apparition and severity of proteinuria. n for controls/TACI‐Fc/BAFFR‐Fc/Apry‐1‐1 were 43/15/15/14. Panels A‐H are from an experiment that was performed once. Panels A‐D, F, and G show mean of each group ± SEM. Symbols represent individual mice. Statistical analysis was performed with one‐way ANOVA followed by Bonferroni comparing controls to each treatment. ns: non‐significant; *p < 0.05; **p < 0.01, ***p < 0.001.

In order to document specific changes induced in mTACI‐Fc‐treated mice, we enumerated antibody‐secreting cells and measured CD138^+^ PCs in the spleen and bone marrow. In the spleen, mTACI‐Fc, and to a lesser extent mBAFFR‐Fc, reduced antibody‐secreting cell numbers and showed a trend toward reduction of CD138^+^ cells (Fig. 2A and B). In the bone marrow, mBAFFR‐Fc and mTACI‐Fc decreased antibody‐secreting cell numbers, with the effect being more pronounced for mTACI‐Fc (Fig. 2C). Only mTACI‐Fc significantly reduced CD138^+^ cells in the bone marrow (Fig. 2D). In the control group, autoantibodies increased or remained high in most animals, while they were stabilized at roughly initial levels in mTACI‐Fc‐treated mice (Fig. 2E). When only considering mice with relatively low autoantibody levels at week 24, i.e. those mice into which large increase of autoantibodies was possible, mTACI‐Fc prevented evolution to high autoantibody levels (Fig. 2F).

### TACI‐Fc decreases renal histopathology even in the presence of autoantibodies

**Figure 3:**
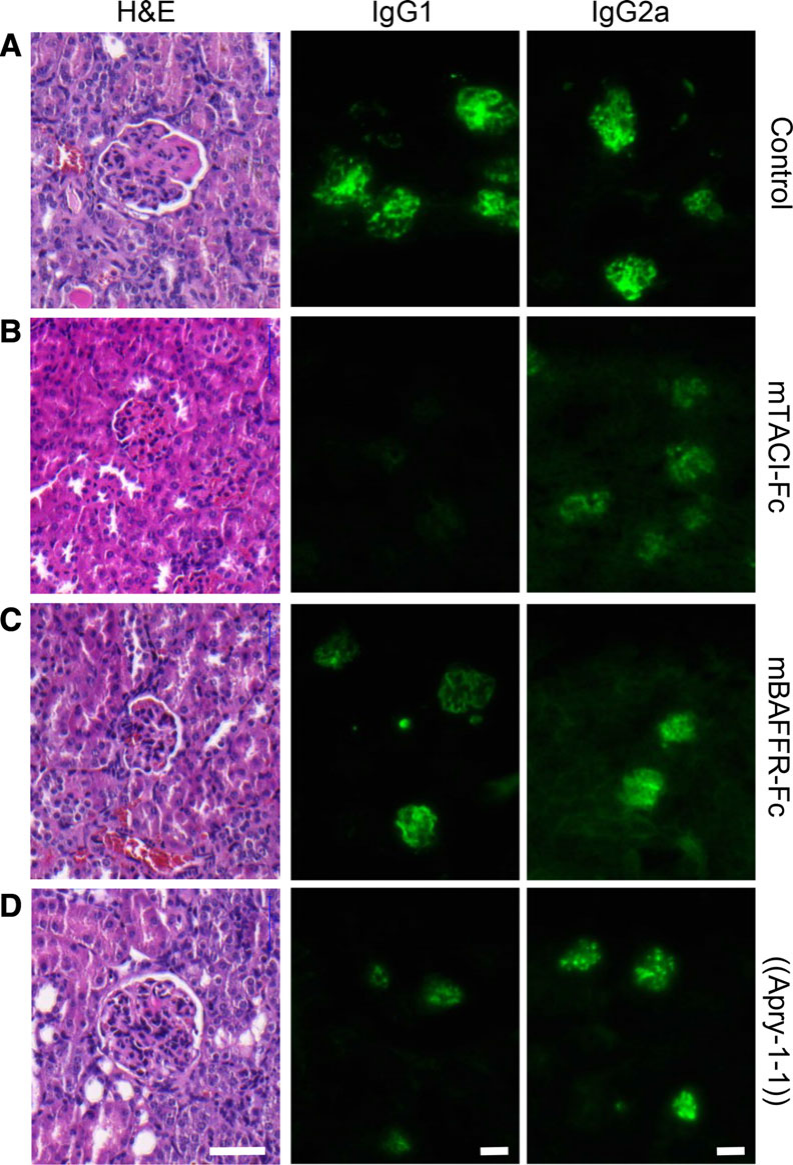
mTACI‐Fc prevents renal immunopathology in NZB/NZW F1 mice. Kidney sections from 37 week‐old NZB/NZW F1 mice treated as indicated and stained with hematoxylin and eosin (H&E), or stained by immunohistochemistry for IgG1 or IgG2a. (A) mFc‐treated (control) mouse. (B) mTACI‐Fc‐treated mouse. (C) mBAFFR‐Fc treated mice. (D) Apry‐1‐1‐treated mouse. Original magnifications × 200 for H&E and × 100 for IHC. Scale bars: 50 μm. Data were used to determine histopathology scores of Fig. 2G. Images are from a single experiment with 15 mice per group (except controls: 45 mice) and are representative of animals with median histopathology scores (see Fig. 2G) without proteinuria (15/15 for mTACI‐Fc) or with proteinuria (28/45 for controls, 7/15 for mBAFFR‐Fc, and 7/15 for Apry‐1‐1).

Upon termination of the study at the age of 37 weeks, kidneys from control mice showed pronounced multifocal to generalized glomerular damage, obliteration of Bowman space, glomerular compression and glomerular sclerosis (Fig. 3A, left panel). In contrast, kidneys from mTACI‐Fc‐treated mice were protected (Fig. 3B) with significantly lower renal histopathology scores (Fig. 2G), while those of mBAFFR‐Fc‐treated mice were intermediate between the control and mTACI‐Fc groups (Fig. 3C and 2G). We next investigated the relationship between autoantibody levels and renal pathology. In the control group, with a few exceptions, high levels of anti‐dsDNA IgG at weeks 24–28 were associated with high proteinuria scores and vice‐versa (Fig. 2H). This correlation was absent in mTACI‐Fc‐treated mice, where proteinuria did not develop even in mice with high anti‐dsDNA titers. A possible intermediate situation was observed in mBAFFR‐Fc treated mice (Fig. 2H).

Taken together, these results indicate that treatment with mTACI‐Fc reduces PC numbers in the bone marrow and slows down autoantibody production, with effects probably extending beyond the mere control of PCs and anti‐dsDNA IgGs, as mice with high and low autoantibody levels were equally protected.

### TACI‐Fc‐induced decrease of PC is not recapitulated with a combination of BAFF and APRIL inhibitors

**Figure 4:**
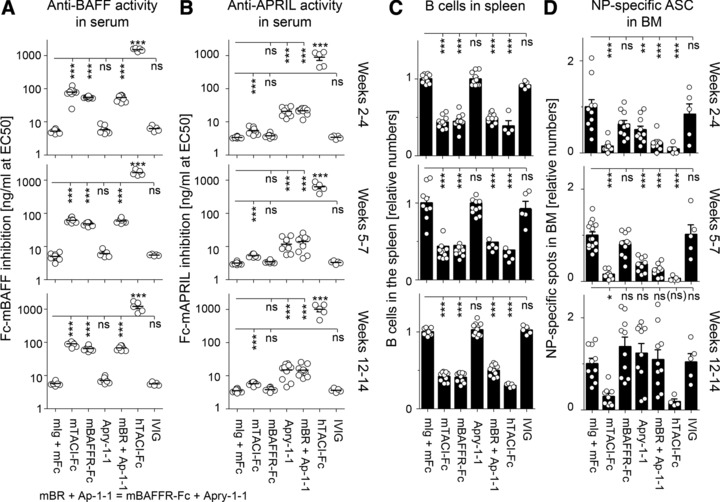
m and hTACI‐Fc, not mBAFFR‐Fc, efficiently deplete bone marrow PC in immunized C56BL/6 mice, while efficacy of Apry‐1‐1 alone or with mBAFFR‐Fc varies as PCs mature. C57BL/6 mice were immunized at 8 weeks of age with NP‐conjugated keyhole limpet hemocyanin precipitated in Alum. 2, 5, or 12 weeks later, mice were treated for two additional weeks, three times a week, with either mTACI‐Fc, hTACI‐Fc, mBAFFR‐Fc, anti‐mAPRIL Apry‐1‐1 or control reagents. Data shown combine results of two independent experiments each with *n* = 5 animals per group (with the exception of hTACI‐Fc and intravenous immunoglobulin (IVIG) that were performed once). (A). Quantification of mouse BAFF‐blocking activity in sera of treated mice at the end of the experiment, using a BCMA:Fas reporter cell line. (B). Same as panel C, but for the quantification of mouse APRIL‐blocking activity. (C). Relative absolute numbers of splenic B cells (CD19^+^/B220^+^), compared to the mean of a control group (mIg + mFc). (D). Relative absolute numbers of bone marrow NP‐specific IgG antibody‐secreting cells (ASC) measured by ELISPOT compared to the mean of a control group. Mice were treated with the indicated inhibitors from weeks 2 to 4 (weeks 2–4), 5 to 7 (weeks 5–7), or 12 to 14 (weeks 12–14) post immunization and analyzed at the end of this 2‐week treatment. All graphs show mean ± SEM. Symbols represent individual mice. Analyses in panels A and B were performed twice with similar results. Statistical analysis was performed with one way ANOVA followed by Bonferroni comparing controls to each treatment (A–D). ns: nonsignificant; ^*^ *p* < 0.05; ^**^ *p* < 0.01; ^***^ *p* < 0.001; (ns): nonsignificant in one way ANOVA, but ^***^ when compared to mIg + mFc with unpaired t‐test. For panels A and B, only hTACI‐Fc is significantly different from all other groups by one way ANOVA (*p* < 0.001). The line of statistical results that is shown in panel A compares controls to inhibitors of similar potency (i.e., excluding the group of hTACI‐Fc) by one‐way ANOVA. For panel B, inhibitors of medium activity (Apry‐1‐1, upper line of statistical results) and of weak activity (mTACI‐Fc, lower line of statistical results) are independently compared to controls by one‐way ANOVA.

How inhibitors impact bone marrow PC survival was addressed in C57BL/6 mice immunized with NP‐conjugated keyhole limpet hemocyanin. Following immunization, mice were rested for 2, 5 or 12 weeks before initiation of a 2‐week treatment, after which time activities of inhibitors were monitored in sera, and levels of splenic B cells and NP‐specific bone marrow PCs were determined. Anti‐BAFF activities in sera were comparable at all time points for mTACI‐Fc, mBAFFR‐Fc given alone and mBAFFR‐Fc given together with Apry‐1‐1 (Fig. 4A). Anti‐APRIL activity of mTACI‐Fc was detectable but surprisingly low, as already observed in the SLE model, while activity of Apry‐1‐1 alone or together with mBAFFR‐Fc was present and higher than that observed for mTACI‐Fc (Fig. 4B). Thus, unlike what was observed in the SLE model, Apry‐1‐1 remained active in immunized C57BL/6 mice. Finally, serum anti‐BAFF and anti‐APRIL activities of hTACI‐Fc were one to two orders of magnitude higher than those of other inhibitors tested (Fig. 4A and B). As anticipated, mTACI‐Fc, hTACI‐Fc and mBAFFR‐Fc all decreased BAFF‐dependent splenic B cells, while Apry‐1‐1 did not (Fig. 4C). For bone marrow PCs, the effect of BAFF and/or APRIL inhibition was dependent on the timing of inhibitor administration post‐immunization. PCs at 2 weeks were decreased upon treatment with mTACI‐Fc, hTACI‐Fc or combined mBAFFR‐Fc/Apry‐1‐1, but not with mBAFFR‐Fc alone, and only to a lesser extent with Apry‐1‐1 alone (Fig 4D, top panel). This suggests that early PCs can survive in response to either BAFF or APRIL, and that both APRIL and BAFF are present in sufficient amounts to support this population. At 5 weeks, mTACI‐Fc and hTACI‐Fc efficiently depleted PCs. Apry‐1‐1, alone or in combination with mBAFFR‐Fc, also significantly reduced PCs, but to a lower extent than TACI‐Fc, while mBAFFR‐Fc showed no efficacy (Fig. 4D, middle panel). This suggests that at week 5, PCs might rely more strongly on APRIL than on BAFF. At week 12, when long‐lived PCs were measured, the variability of the assay was relatively high, and only mTACI‐Fc treatment significantly decreased PCs. PCs were also low in all hTACI‐Fc‐treated animals (but statistically non‐significant because of the relatively low number of mice in this group) (Fig 4D, bottom panel). Single BAFF or APRIL inhibitors, alone or in combination, did not decrease PCs at that stage. Especially, in the mBAFFR‐Fc/Apry‐1‐1 group, despite homogenous anti‐BAFF and anti‐APRIL activities (Fig. 4A and B), numbers of PCs were very heterogeneous with a mean not different from that of the control group (Fig. 4D, bottom panel). We conclude that long‐lived PCs are decreased by TACI‐Fc, but not by separate inhibitions of BAFF and APRIL.

Taken together, these observations confirm that TACI‐Fc, but not BAFFR‐Fc, can inhibit bone marrow PC, including long‐lived ones 40. They further indicate that combined inhibition of BAFF and APRIL with two specific inhibitors does not always recapitulate the action of TACI‐Fc on long‐lived PCs, and suggest that PCs at different stages of maturation may rely on different combinations of survival cytokines.

## Discussion

This study was designed to compare single versus dual pharmacological inhibitions of BAFF and APRIL in a mouse model of SLE and in a hapten/carrier immunization model. We used an anti‐mAPRIL single‐chain monoclonal antibody for specific APRIL inhibition, mBAFFR‐Fc for single BAFF inhibition and mTACI‐Fc, the mouse equivalent of atacicept currently being developed as a treatment for SLE 41, for dual BAFF and APRIL inhibition.

Quantification of inhibitor activity in sera of experimental animals was informative. First, most autoimmunity‐prone NZB/NZW F1 mice mounted a robust neutralizing antibody response to the anti‐APRIL antibody Apry‐1‐1 but this anti‐drug response was not detected after 2 weeks of treatment in C57BL/6 mice in the hapten/carrier immunization model. Second, the BAFF‐blocking activity of mBAFFR‐Fc was comparable to that of mTACI‐Fc in vitro and in vivo in both animal models used. Third, in terms of APRIL‐neutralizing activity, hTACI‐Fc and Apry‐1‐1 behaved identically in vitro, but the later displayed a 30‐ to 300‐fold lower activity in vivo in C57BL/6 mice, in the absence of a detectable anti‐drug response. Fourth, hTACI‐Fc inhibited mBAFF and mAPRIL in vitro and in vivo. In comparison, after normalization to anti‐mBAFF activity, mTACI‐Fc was five‐fold less active against mAPRIL in in vitro assays. The molecular basis for this intrinsic partial deficit of mTACI‐Fc toward mAPRIL inhibition is unknown. The anti‐mAPRIL activity of mTACI‐Fc in vivo dropped even further, possibly because a fraction of this close‐to‐limiting activity became occupied by endogenous ligands. All in all, mBAFFR‐Fc and mTACI‐Fc inhibited mBAFF similarly in all experiments, while Apry‐1‐1 could inhibit more mAPRIL than mTACI‐Fc in C56BL/6 mice but was inactivated in autoimmune mice. The latter result might be explained by the presence of human sequences in Apry‐1‐1.

Previous studies conducted in NZB/NZW F1 and related mouse models of lupus have led to the general conclusion that TACI‐Fc and BAFFR‐Fc were equally efficient to prevent lupus when administered as single agents before development of proteinuria, or to arrest or even reverse further disease development when administered together with CTLA4‐Fc, a T‐cell inhibitor, after onset of proteinuria 13, 28, 29, 30. This, together with the modest effect of anti‐APRIL therapy observed in this model 32, indicated a predominant pathological role of BAFF in mouse SLE, with no or little contribution of APRIL. In agreement with others, we found that TACI‐Fc as single agent strongly impaired disease progression, even though we initiated treatment at a later time point, when autoantibodies were already present and proteinuria about to appear. However, we observed that BAFFR‐Fc was far less efficient than TACI‐Fc in arresting SLE, which, at first sight, contrasts with previous publications. It is, however, noteworthy that even in our study, mBAFFR‐Fc had signs of efficacy when renal pathology was assessed (Fig. 2G). Discrepancies observed for the efficacy of BAFFR‐Fc might reflect different experimental conditions. Housing conditions or other variables could differently modulate kinetics and penetrance of disease. Also, timing of treatment was different in our study compared to the most similar one performed in NZB/NZW F1, where treatment was initiated in a prophylactic manner at weeks 18–20, before declared autoimmunity (see Fig. 1A) 30, 31. In this prophylactic study, appearance of proteinuria in mBAFFR‐Fc‐treated mice was delayed by 24 weeks (50% proteinuria ≥300 mg/dL at week 55 instead of week 31 in controls) and death was remarkably delayed (50% survival at week 35 in untreated, versus week 65 in treated) 30. Other differences were the dose and mode of inhibitor administration: mBAFFR‐Fc was expressed from a single administration of adenovirus, resulting in very high expression during the first 2 weeks reaching up to 500 μg/mL in serum before declining to about 20 μg/mL after 5 weeks; it cannot be fully excluded that mBAFFR‐Fc at high concentrations might have cross‐reacted to partially or entirely neutralize comparatively very low levels of endogenous APRIL. Indeed, in a different experimental system, glycolipid‐anchored mBAFFR, but not hBAFFR, could specifically cross react with APRIL 42. In another study performed in NZB/NZW F1 mice, BAFFR‐Fc was tested at week 24 (100 μg ip, three times a week, until week 29), long before proteinuria became visible (50% of untreated mice had proteinuria at week 42). The species of BAFFR‐Fc used was not reported and TACI‐Fc was not tested in this study, but there was a positive impact of treatment on proteinuria and survival (40% in untreated versus 100% in treated at week 52) 28.

In control groups of NZB/NZW F1 mice, we found that autoantibody levels at the beginning of disease often correlated with a proteinuria score at the end of the disease (Fig. 2H). This score took into account timing of the first detection of proteinuria and average proteinuria levels during the last 4 weeks of the experiment. This correlation was broken in TACI‐Fc‐treated mice, where even mice with high autoantibody levels did not develop proteinuria. The observation that TACI‐Fc treatment decreased PCs in the bone marrow and slowed down autoantibody formation cannot explain alone protection observed in mice with high autoantibody levels and suggest involvement of other mechanisms. It has been shown in the NZM 2410 non‐inflammatory SLE model that immune complex deposition in kidney could be uncoupled from renal pathology in adenovirus‐mBAFFR‐Fc treated mice 29. Indeed, BAFF inhibition prevented renal pathology, reduced splenic B cells and induced a small spleen size, but did not prevent immune complex deposition in kidneys. Small spleens contain fewer numbers of activated CD4 T cells and CD11b macrophages/dendritic cells, which may account for the observed decreased activation of resident dendritic cells in kidney, decreased activation of renal endothelial cells and preservation of podocytes, which are part of the glomerular filtration barrier 29. Thus, autoantibodies and immune complexes could collaborate with other (inflammatory) mediators to damage kidney. TACI‐Fc may affect both processes to exert its protective effect.

Here we observe that, at least in the specific conditions of our experiment, mTACI‐Fc was active when mBAFFR‐Fc was not or little. This could be due to APRIL inhibition. For example, APRIL‐deficient mice displayed reduced IL‐17 secretion and had reduced collagen‐induced arthritis manifestations 43. But it could also be due to a distinct activity of TACI‐Fc. The question of whether APRIL inhibition can explain the protective effect of TACI‐Fc will be clarified when reagents that specifically block mouse APRIL but that are not neutralized in autoimmune mice become available.

With regard to BAFF and/or APRIL requirement of long‐lived bone marrow PCs in immunized, non‐autoimmune mice, a previous, elegant study has compared the outcome of treatments with TACI‐Fc or BAFFR‐Fc in wild type and APRIL‐ko mice. TACI‐Fc always decreased long‐lived PCs, but BAFFR‐Fc did so only in APRIL‐ko mice 40, leading to the conclusion that long‐lived PCs use either BAFF and/or APRIL as survival factors in vivo. We used a pharmacological inhibitor of APRIL instead of APRIL‐ko mice and got identical conclusions for newly generated bone marrow PCs, but not for long‐lived ones that were sensitive to TACI‐Fc, but not to BAFFR‐Fc/anti‐APRIL administered together. It is an intriguing possibility that these results may reflect a role of heteromers of BAFF and APRIL 4, 5, 6 in the biology of PCs. Indeed, an anti‐APRIL antibody may not inhibit heteromers of BAFF and APRIL, while no heteromer can form in APRIL‐ko mice. The development of reagents that can specifically block mouse APRIL/BAFF heteromers, would help assessing the putative role of heteromers in PC biology.

## Materials and methods

### Mice and spontaneous SLE model

Protocols had been legally approved and experiments were conducted in accredited facilities in accordance with Merck KGaA Institutional Animal Care and Use Committee (IACUC) guidelines and compliant with regulations set by the German animal protection law, enforced by the Regierungspräsidium, Darmstadt, Hessen, Germany (authorizations DA4/205 and DA4/222). Mice were housed in specific pathogen‐free, barrier facilities at all times.

Female F1 hybrid mice NZBWF1/J (the progeny of female NZB/BlNJ and male NZW/LacJ) were purchased from The Jackson Laboratory. Blood and urine samples were collected for longitudinal monitoring of autoantibodies and urine protein content, starting at 16 weeks of age. C57BL/6 mice were purchased from Charles River Laboratories. MRL^lpr/lpr^ mice were purchased from Harlan Laboratories.

### Antibodies and recombinant proteins

Fc‐mBAFF, and Fc‐mAPRIL were produced essentially as described (reviewed in Schneider, et al 2014 44). A single‐chain human neutralizing anti‐mouse APRIL antibody fused to mouse IgG2b (Apry‐1‐1) was obtained from Adipogen (Epalinges, Switzerland). hTACI (aa 31–110) ‐ hIgG1 Fc (aa 245–470, L258E, A353S, P354S) (atacicept, 41) was obtained from Merck KGaA. mTACI (aa 2–82) ‐ mIgG2 Fc (L235E, E318A, K320A, K322A), mBAFFR (aa 2–76) ‐ mIgG2c Fc (L235E, E318A, K320A, K322A), mouse IgG2c Fc (L235E, E318A, K320A, K322A) and mouse IgG2b (clone MCP‐11) were originally from Zymogenetics and provided by Merck KGaA. Mutations in the Fc domains disrupt binding to Fc receptors and complement, as described 40.

### Immunizations and treatments

NZB/NZW F1 mice were randomly assigned to experimental groups and received test articles starting at week 25, when the majority of animals had developed significantly increased anti‐dsDNA autoantibody titers. mTACI‐Fc, mBAFFR‐Fc, anti‐mouse APRIL Apry‐1‐1, mouse Fc fragment (mFc), or mouse isotype control (mIg) were administered i.p. at 5 mg/kg, three times per week on a Monday/Wednesday/Friday schedule for 12 weeks. Serum and urine samples were collected for anti‐dsDNA antibodies (by ELISA) and urinary protein to creatinine ratio (UPCR) (measured by ADVIA 1800) determination, respectively, on the days indicated. Proteinuria was defined as when UPCR was ≥ 3. A proteinuria score was defined as the average UPCR at weeks 34, 35, 36, and 37 divided by the number of proteinuria‐free weeks since week 22. For mice sacrificed before week 34 because of proteinuria, the value of the last UPCR measure was used to calculate the proteinuria score.

For hapten/carrier immunizations, 6 to 8 weeks‐old mice were immunized i.p. with 100 μg of NP‐conjugated keyhole limpet hemocyanin adsorbed to alum (Imject^®^, Pierce) in a volume of 200 μL. After immunization, mice were treated three times a week for 2 weeks with test articles or controls at 5 mg/kg, starting at week 2, 5, or 12 post‐immunization. Mice were then sacrificed and tissues harvested.

### Histology

Sections of formalin‐fixed kidneys harvested from NZB/NZW F1 mice post mortem were stained with hematoxylin and eosin (H&E) and were blindly assessed by light microscopy for inflammation, glomerular damage and sclerosis. Glomerular activity (hypercellularity, necrotizing lesions, karyorrhexis, cellular crescents, hyaline deposits), tubulointerstitial activity (interstitial cellular infiltration, tubular cell necrosis), chronic glomerular pathology (glomerulosclerosis, fibrous crescents), and chronic tubulointerstitial pathology (tubular atrophy, interstitial fibrosis) were assessed as follows and subjectively scored on a 0–4 scale, for a maximum composite score of 12: Inflammation: Grade 1, mild focal interstitial inflammation; grade 2, multifocal areas of mild interstitial inflammation; grade 3, moderate multifocal interstitial inflammation; grade 4, significant multifocal interstitial inflammation. Glomerular damage: Grade 1, initial glomerular lesions; initial lesions are characterized by increased cellular components in single or few glomeruli, cellular proliferation and basement membrane thickening; grade 2, multifocal areas of glomerular lesions; grade 3, multifocal areas of glomerular lesions with significant damage characterized by proliferation of epithelial cells of capsule of Bowman with compression of glomerular capillaries; grade 4, pronounced multifocal to generalized glomerular damage (*i.e*. grade 3 plus obliteration of Bowman space, glomerular compression and hyalinosis). Glomerular sclerosis: Grade 1, focal mild glomerular sclerosis; grade 2, multifocal mild glomerular sclerosis; grade 3, severe focal glomerular sclerosis; grade 4, severe multifocal glomerular sclerosis.

### Determination of anti‐dsDNA titers

ELISA plates were coated with 10 μg of calf thymic DNA (Sigma) in 0.1 M Na carbonate buffer pH 9.6, blocked, exposed to serial dilutions of sera, and revealed with horseradish peroxidase‐coupled goat anti‐mouse IgG antibodies and 3,3′,5,5′‐tetramethylbenzidine (Sigma‐Aldrich). Anti‐dsDNA antibody levels were calculated as arbitrary units compared to a pooled standard serum from 22‐week‐old MRL^lpr/lpr^ mice.

### Immunofluorescence

Tissues were snap‐frozen and 5 μm sections were fixed with methanol prior to staining for 1 h at room temperature with FITC‐conjugated anti‐mouse IgG1 or IgG2a (Southern Biotech). Slides were analyzed with an Axio Imager 2 (Zeiss) used at 100× magnification. Images were captured using a CCD camera (AXIOCam MRm Zeiss).

### Detection of antibody‐secreting cells by ELISPOT

ELISPOT plates (Millipore) were coated with 10 μg/mL of goat anti‐mouse IgG (Calbiochem) or 10 μg/mL of NP‐BSA (Biosearch Technologies) in 0.1 M carbonate buffer pH 9.6 and washed with PBS. Serial dilutions of bone marrow cell or splenocytes were incubated for 5 h, followed by washing with PBS and incubation with secondary horse radish peroxydase‐conjugated anti‐mouse IgG (Sigma). After washing, plates were developed with AEC staining Kit (Sigma) until spots became visible with bare eyes, then washed with water and dried. Spots were counted with an ELISPOT reader (AID)

### Flow cytometry

Spleens were harvested, and splenocytes were prepared according to conventional procedures, including a red blood cell lysis step in 156 mM NH_4_Cl, 10 mM NaHCO_3_, 0.1 mM EDTA, pH 7.3 (ACK lysis buffer) for 10 min at room temperature. Splenocytes were incubated in Fc‐block (anti CD16/32) then stained with CD19‐APC‐Cy7 (Biolegend), B220 pacific orange (Invitrogen) and CD138 APC (BD Bioscience) antibodies. Cells were analyzed using a FACS Canto flow cytometer (BectonDickinson) and FlowJo software (TreeStar, Ashland, OR).

### In vitro cytotoxicity assays

A Fas‐deficient reporter cell line Jurkat JOM2 BCMA:Fas‐2309 cl56 was generated as previously described 44. These cells are more sensitive (three‐ to 10‐fold) than the previously reported Jurkat BCMA:Fas‐2309 cl13 cells 45. Cells were exposed overnight to titrated amounts of Fc‐mAPRIL or Fc‐mBAFF, in the presence of fixed amounts of inhibitor‐containing sera in a total volume of 100 μL of RPMI medium supplemented with 10% fetal calf serum and antibiotics, after which time cell viability was measured by the phenazine methosulfate / 3‐(4,5‐dimethylthiazol‐2‐yl)‐5‐(3‐carboxymethoxyphenyl)‐2‐(4‐sulfophenyl)‐2H‐tetrazolium (PMS/MTS) assay essentially as described 44. Fc‐mAPRIL was used at a first final concentration of 40 ng/mL, with 12 three‐fold dilutions (except when measuring hTACI‐Fc activity: first final concentration of 500 ng/mL, with four‐fold dilutions). Fc‐mBAFF was used at a first final concentration of 100 ng/mL, with three‐fold dilutions. Sera of treated mice were tested at a final dilution of 1/300. EC_50_ were determined for each titration curve using the “log(agonist) versus normalized response – Variable slope” function of the Prism software. For the measure of anti‐Apry‐1‐1 neutralizing antibody response, Jurkat BCMA:Fas‐2309 cl13 were exposed to a fixed, lethal concentration of Fc‐mAPRIL (1 ng/mL) in the presence of titrated amounts of fresh Apry‐1‐1 (to inhibit Fc‐mAPRIL) and of a fixed dilution of sera from control‐ or Apry‐1‐1‐treated NZB/NZW F1 mice.

### Statistics

Statistical analyses were performed with the Prism software, as described in the figure legends.

## Conflict of interest

P.H. and H.H. are employees of Merck KGaA, Germany. P.S. is supported by a research grant from EMD Serono, a subsidiary of Merck, KGaA. M.V. and J.N. declare no commercial or financial conflict of interest.

**Abbreviations**
APRIL: A proliferation‐inducing ligand
BAFF: B cell activating factor of the TNF family
BAFFR: BAFF receptor
BCMA: B cell maturation antigen
Fc: fragment crystallizable of an antibody
NP: 4‐hydroxy‐3‐nitrophenylacetyl hapten
PC: plasma cell
TACI: transmembrane activator and CAML [calcium‐modulator and cyclophilin ligand] interactor
UPCR: urinary protein to creatinine ratio

## Supporting information

Supporting Information Figure 1. Characterization of mAPRIL‐neutralizing agents.
Supporting Information Figure 2. Gating strategies for FACS analyses.

Supporting Information
