# Supplementary material for: A mouse model of systemic lupus erythematosus responds better to soluble TACI than to soluble BAFFR, correlating with depletion of plasma cells
Source: Eur J Immunol. 2017 Apr 24;47(6):1075–85. doi: 10.1002/eji.201746934 (PMC5518279; doi:10.1002/eji.201746934)
Supplement: Supplementary file 1 — Supporting Information Figure 1. Characterization of mAPRIL‐neutralizing agents. Supporting Information Figure 2. Gating strategies for FACS analyses. [file EJI-47-1075-s001.pdf]

European Journal of Immunology

Supporting Information
for

DOI 10.1002/eji.201746934

Philipp Haselmayer, Michele Vigolo, Josquin Nys, Pascal Schneider and Henry Hess

A mouse model of systemic lupus erythematosus responds better to soluble TACI than to soluble BAFFR, correlating with depletion of plasma cells

© 2017 The Authors. *European Journal of Immunology* published by WILEY-VCH Verlag GmbH & Co. KGaA, Weinheim.

Supporting information

A mouse model of systemic lupus erythematosus responds better to soluble TACI than to soluble BAFFR, correlating with depletion of plasma cells.

Philipp Haselmayer, Michele Vigolo, Josquin Nys, Pascal Schneider and Henry Hess

Abbreviations: Flag: octapeptide tag with sequence DYKDDDDK. 293T cells: human embryonic kidney-derived cell line that contains the SV40 Large T-antigen. GPI: glycosyl-phosphatidylinositol.

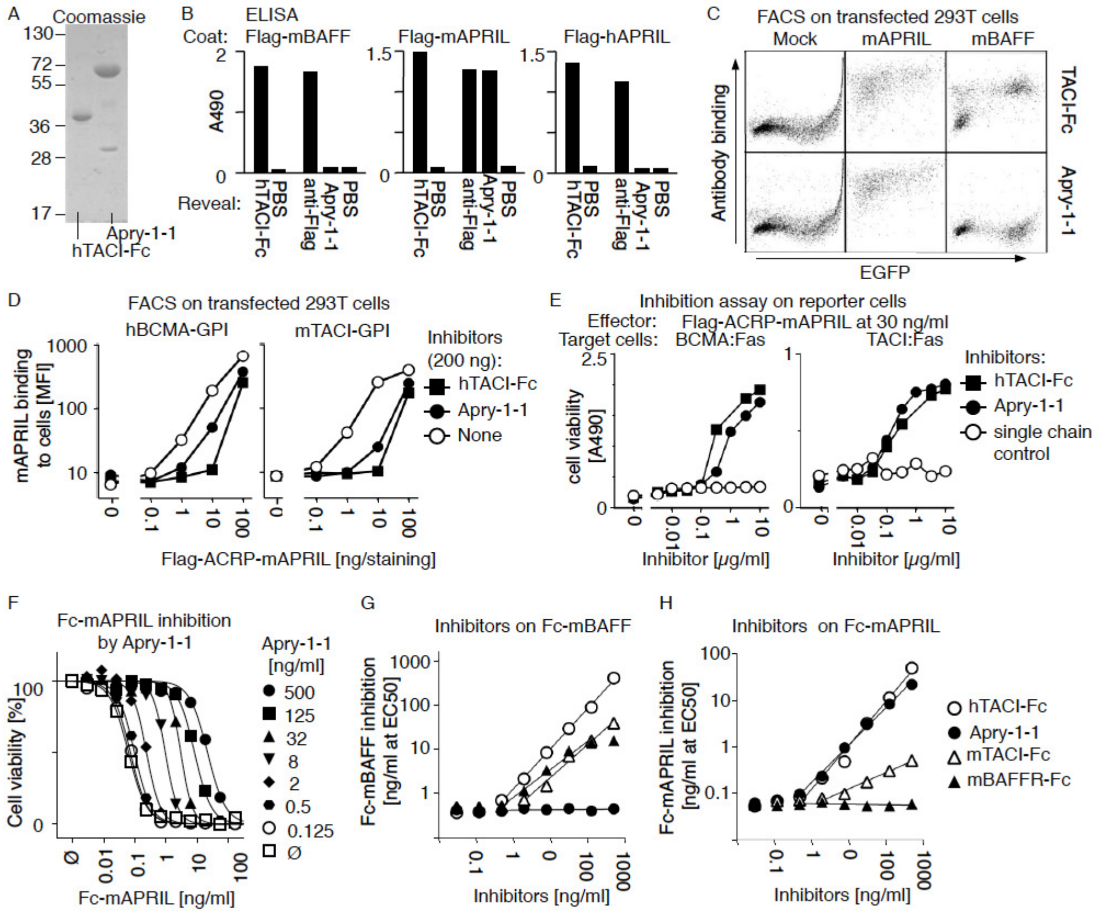

Supporting Figure 1. Characterization of mAPRIL-neutralizing agents.
TACI-Fc and the recombinant anti-mouse APRIL Apry-1-1 were compared in a panel of tests for their ability to bind and inhibit BAFF and/or APRIL in vitro
A. Coomassie blue staining of 10 µg/lane of TACI-Fc and Apry-1-1.
B. Apry-1-1 specifically recognizes mAPRIL. Flag-mBAFF, Flag-ACRP-mAPRIL or Flag-ACRP-hAPRIL were coated in an ELISA plate and revealed with either hTACI-Fc, an anti-Flag

antibody or Apry-1-1 reagents (or PBS as a control) as indicated, followed by appropriate horse radish peroxydase-coupled anti-human or anti-mouse antibodies for hTACI-Fc and Apry-1-1, respectively.

C. Apry-1-1 specifically recognizes native mAPRIL. 293T cells were co-transfected with an enhanced green fluorescent protein (EGFP) tracer and with non-cleavable forms of mouse APRIL or BAFF. EGFP expression on the X-axis correlates with mAPRIL or mBAFF expression. Cells were stained by FACS with TACI-Fc or Apry-1-1, followed by appropriate phycoerythrin-coupled secondary reagents. Scattergrams show four orders of magnitude fluorescence on a logarithmic scale on both axes. Binding of hTACI-Fc or Apry-1-1 is revealed by an increased fluorescence on the Y axis (antibody binding).

D. Apry-1-1 prevents the binding of mAPRIL to BCMA and TACI. 293T cells transfected with glycosyl-phosphatidylinositiol (GPI)-anchored BCMA or TACI were stained with titrated amounts of Flag-ACRP-mAPRIL either alone (none) or in the presence of the indicated fixed amount of TACI-Fc or Apry-1-1. The binding of Flag-ACRP-mAPRIL to BCMA or TACI was monitored using appropriate phycoerythrin-coupled secondary reagents and expressed as mean fluorescence intensity (MFI).

E. Apry-1-1 specifically protects BCMA and TACI reporter cells from mAPRIL-mediated death. Reporter cells expressing the chimeric receptors BCMA:Fas or TACI:Fas activate a surrogate Fas apoptotic pathway in response to APRIL. Reporter cells had been exposed to a lethal dose of Flag-ACRP-mAPRIL, in the presence or absence of increasing concentrations of TACI-Fc or Apry-1-1. The presence of viable cells, monitored with a cell viability assay, indicates that mAPRIL activity has been neutralized.

F. BCMA:Fas-2309 cl13 reporter cells were exposed to titrated amounts of Fc-mAPRIL in the presence of fixed concentrations of Apry-1-1, as indicated. Cell viability was monitored to determine EC_{50} of Fc-mAPRIL activity in the presence of various concentrations of Apry-1-1. These data were used to draw the curve of Apry-1-1 in panel H.

G, H. EC_{50} of Fc-mBAFF (panel G) or Fc-mAPRIL (panel H) on BCMA:Fas-2309 cl13 reporter cells plotted as a function of inhibitor concentration. Linear regression curves were determined for EC_{50} values above the lower limit of quantification.

In vitro characterization of a function-blocking anti-mouse APRIL antibody – As BCMA participates to survival and longevity of PCs in the bone marrow [1], and displays a higher affinity for APRIL than for BAFF [2], APRIL is likely to transmit survival signal to PCs. We thus characterized an anti-APRIL antibody suitable for pharmacological inhibition of mouse APRIL. This reagent, Apry-1-1, is a single chain monoclonal antibody dimerized by fusion to the Fc portion of mouse IgG2b. The analysis of Apry-1-1 protein by Coomassie blue, or of hTACI-Fc as a control, revealed single bands of expected sizes with the presence of minor unidentified degradation products or contaminants in the case of Apry-1-1 (Suppl. Fig. 1A). Both reagents recognized Flag-mAPRIL coated in an ELISA plate, while, as expected, only hTACI-Fc additionally recognized Flag-mBAFF and Flag-hAPRIL (Suppl. Fig. 1B). Similar results were obtained when uncleavable forms of mAPRIL and mBAFF were expressed at the surface of transfected 293T cells, further indicating that Apry-1-1 and hTACI-Fc also recognize native conformations of their targets (Suppl. Fig. 1C). These results indicate that hTACI-Fc binds to BAFF and APRIL, while Apry-1-1 is specific for mouse APRIL. Tests were then performed

to assess the inhibitory potential of Apry-1-1. 293T cells were transfected with glycolipid-anchored forms of BCMA or TACI to ensure efficient surface expression of the receptors [3], and stained with increasing concentrations of Flag-ACRP-mAPRIL. When stainings were performed in the presence of Apry-1-1 or TACI-Fc, the binding of mouse APRIL was decreased or even abolished at sufficient inhibitor to ligand ratio (20 and 200 mass excess of hTACI-Fc, respectively Apry-1-1, over mAPRIL), indicating that the inhibitors can interfere with the binding of mouse APRIL to its receptors (Suppl. Fig. 1D).

The functionality of anti-APRIL reagents was assessed in reporter cell lines expressing the extracellular domains of BCMA [4] or of TACI cysteine-rich domains 1 and 2 [5] fused to the transmembrane and intracellular domains of the death receptor Fas, so that engagement of these chimeric receptors with APRIL results in transmission of apoptotic signals and cell death. These cells were totally killed by 30 ng/ml of Flag-ACRP-mAPRIL, an active recombinant form of APRIL, but were specifically rescued in a dose-dependent manner by both hTACI-Fc or Apry-1-1 with EC50 of around 0.3 µg/ml (Suppl. Fig. 1E).

In conclusion, these experiments suggest that the single-chain anti-mouse APRIL antibody is capable of binding and inhibiting mouse APRIL in a variety of tests in vitro and compares favorably with TACI-Fc in terms of mouse APRIL inhibition.

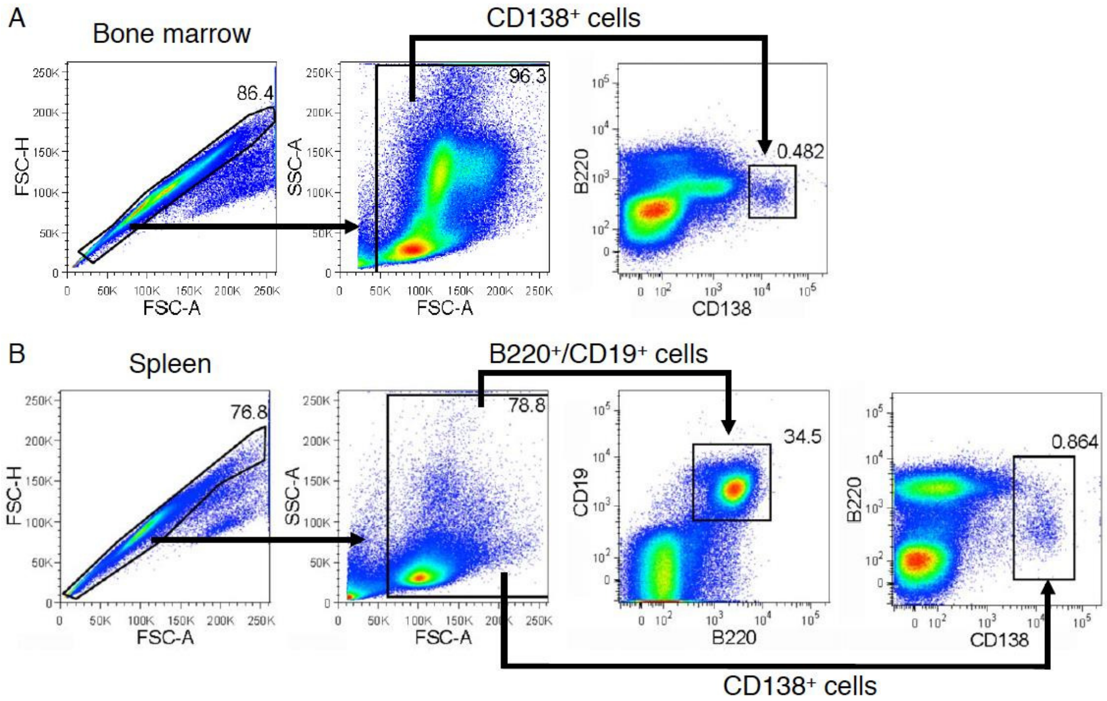

Supporting Figure 2. Gating strategies for FACS analyses.
Examples shown are from NZB/NZW F1 mice. Identical strategies were applied to C57BL/6 mice.
A. Quantification of CD138$^+$ cells in the bone marrow.
B. Quantification of CD138$^+$ cells and B220$^+$/CD19$^+$ cells in the spleen.

Supporting methods

Antibodies and recombinant proteins – Flag-mBAFF, Flag-ACRP-mAPRIL and Flag-ACRP-hAPRIL were produced essentially as described (reviewed in [6]). Mouse IgG1 anti-Flag M2 (± biotinylation) was from Sigma.

ELISA for the detection of Flag-APRIL – Nunc Maxisorp 96-wells immunoplates were coated with Flag-mBAFF, Flag-ACRP-mAPRIL or Flag-ACRP-hAPRIL at 1 µg/ml in PBS. After saturation of wells, proteins were revealed with hTACI-Fc, Apry-1-1 or anti-Flag M2 at 2 µg/ml, followed by appropriate horse radish peroxydase-coupled secondary reagents and o-phenylenediamine (OPD) substrate (Sigma). Reactions were stopped with HCl, and absorbance monitored at 490 nm.

Flow cytometry – 293T cells were co-transfected by the calcium phosphate method with an enhanced green fluorescent protein (EGFP) tracer expression plasmid and plasmids coding for non-cleavable forms of mouse BAFF and mouse APRIL, stained with hTACI-Fc or Apry-1-1 at 5 µg/ml, followed by appropriate phycoerythrin-coupled secondary reagents essentially as described [7]. 293T cells transfected with glycolipid-anchored forms of human BCMA or mouse TACI were stained in 50 µl with 100, 10, 1, 0.1 or 0 ng/staining of Flag-ACRP-mAPRIL in the presence or absence of 200 ng/staining of TACI-Fc or Apry-1-1. Bound Flag-ACRP-APRIL revealed with biotinylated M2 and phycoerythrin-coupled streptavidin was quantified as mean fluorescence intensity in cells expressing medium levels (MFI 100 to 1000) of EGFP essentially as described [6].

In vitro cytotoxicity assays – The reporter cell line Jurkat BCMA:Fas-2309 cl13 was as previously described [4]. Reporter cells Jurkat JOM2 TACI:Fas-2454 cl111 were generated according to a published protocol [6]. Receptor:Fas reporter cells were exposed to 30 ng/ml of Flag-ACRP-mAPRIL in the presence or absence of graded amounts of hTACI-Fc or Apry-1-1. After overnight incubation, cell viability was monitored with the PMS/MTS cell viability assay as described [6]. In other experiments, Jurkat BCMA:Fas-2309 cl13 were exposed to Fc-mAPRIL (500 ng/ml and three-fold dilutions for strong inhibitors, or 20 ng/nl and two-fold dilutions for weak inhibitors) or Fc-mBAFF (400 ng/ml and two-fold dilutions) in the presence of 500, 125, 32, 8, 2, 0.5, 0.125 or 0 ng/ml of hTACI-Fc, Apry-1-1, mTACI-Fc or mBAFFR-Fc. EC_{50} were determined for each titration curve using the "log(agonist) vs. normalized response -- Variable slope" function of Prism.

References for data in the supporting information

1 **O'Connor, B. P., Raman, V. S., Erickson, L. D., Cook, W. J., Weaver, L. K., Ahonen, C., Lin, L., Mantchev, G. T., Bram, R. J. and Noelle, R. J.,** BCMA is essential for the survival of long-lived bone marrow plasma cells. J. Exp. Med. 2004. **199**: 91-97.

2 **Bossen, C. and Schneider, P.,** BAFF, APRIL and their receptors: structure, function and signaling. Semin Immunol 2006. **18**: 263-275.

3 **Bossen, C., Ingold, K., Tardivel, A., Bodmer, J. L., Gaide, O., Hertig, S., Ambrose, C., Tschopp, J. and Schneider, P.,** Interactions of tumor necrosis factor (TNF) and TNF receptor family members in the mouse and human. J Biol Chem 2006. **281**: 13964-13971.

4 **Bossen, C., Cachero, T. G., Tardivel, A., Ingold, K., Willen, L., Dobles, M., Scott, M. L., Maquelin, A., Belnoue, E., Siegrist, C. A., Chevrier, S., Acha-Orbea, H., Leung, H., Mackay, F., Tschopp, J. and Schneider, P.,** TACI, unlike BAFF-R, is solely activated by oligomeric BAFF and APRIL to support survival of activated B cells and plasmablasts. Blood 2008. **111**: 1004-1012.

5 **Kimberley, F. C., van der Sloot, A. M., Guadagnoli, M., Cameron, K., Schneider, P., Marquart, J. A., Versloot, M., Serrano, L. and Medema, J. P.,** The design and characterization of receptor-selective APRIL variants. J Biol Chem 2012. **287**: 37434-37446.

6 **Schneider, P., Willen, L. and Smulski, C. R.,** Tools and techniques to study ligand-receptor interactions and receptor activation by TNF superfamily members. Methods Enzymol 2014. **545**: 103-125.

7 **Bossen, C., Tardivel, A., Willen, L., Fletcher, C. A., Perroud, M., Beermann, F., Rolink, A. G., Scott, M. L., Mackay, F. and Schneider, P.,** Mutation of the BAFF furin cleavage site impairs B-cell homeostasis and antibody responses. Eur J Immunol 2011. **41**: 787-797.

Suporting Table 1. **Plasmids used in this study.**

**Plasmid**	**Designation**	**Protein encoded**	**Vector**
ps015	Empty vector	None	PCR3
ps515	EGFP	Enhanced green fluorescent protein	PCR3
ps657	Flag-mBAFF	HA signal-Flag-GPGQVQLQVD-mBAFF (aa 127-309)	PCR3
ps813	hBAFF N-mAPRIL	hBAFF (aa 1-132)-LQ-mAPRIL (aa96-240)	PCR3
px1003	hBAFF N-mBAFF	hBAFF (aa 1-132)-LQVD-mBAFF (aa128-309)	PCR3
ps1153	Fc-mAPRIL	HA signal-LD-hIgG1 (aa 245-470)-RSPQPQPKPQPKPEPEGSLQ-mAPRIL (aa 96-240)	PCR3
ps1159	mTACI-GPI	Ig signal-LE-mTACI (aa 2-78)-AAAVD-hTRAILR3 (aa 157-269)	PCR3
ps1219	Fc-mBAFF	HA signal-LD-hIgG1 (aa 245-470)-RSPQPQPKPQPKPEPEGSLQVD-mBAFF (aa 127-309)	PCR3
ps1309	Flag-ACRP-mAPRIL	HA signal-Flag-GPGQVQLH-mACRP30 (aa 18-111)-LQ-mAPRIL (aa 106-240)	PCR3
ps1377	pMSCV-puro	Modified pMSCV-puro (Clonetech) with HindIII-BglII-EcoRI-NotI-XhoI-HpaI-ApaI cloning sites	ps1377
ps1467	hBCMA-GPI	Ig signal-VQCEVKLVPRGS-hBCMA (aa 2-54)-VD-hTRAILR3 (aa 157-269)	PCR3
ps1623	Flag-ACRP-hAPRIL	HA signal-Flag-GPGQVQLH-mACRP30 (aa 18-111)-LQ-hAPRIL (aa 98-233)	PCR3
ps2309	hBCMA:Fas	Ig signal-VQCEVKLVPRGS-hBCMA (aa 2-54)-VD-hFas (aa 169-335)	ps1377
ps2455	hTACI:Fas	HA signal-L-hTACI (aa 67-118)-VD-hFas (aa 169-335)	ps1377

Flag = DYKDDDDK HA signal=MAIIYLILLFTAVRG Ig signal=MNFGFSLIFLVLVLKG
