# Supplementary material for: A mouse model of systemic lupus erythematosus responds better to soluble TACI than to soluble BAFFR, correlating with depletion of plasma cells
Source: Eur J Immunol. 2017 Apr 24;47(6):1075–85. doi: 10.1002/eji.201746934 (PMC5518279; doi:10.1002/eji.201746934)
Supplement: Supplementary file 2 — Supporting Information [file EJI-47-1075-s002.pdf]

European Journal of Immunology

A mouse model of systemic lupus erythematosus responds better to soluble TACI than to soluble BAFFR, correlating with depletion of plasma cells

Philipp Haselmayer, Michele Vigolo, Josquin Nys, Pascal Schneider and Henry Hess

Correspondence: Dr. Pascal Schneider, Department of Immunopharmacology, Immunology Translational Innovation Platform, Merck KGaA, Darmstadt, Germany.

Review Timeline:	Submission date:	09-Jan-2017
	First Editorial decision:	23-Jan-2017
	First revision received:	28-Jan-2017
	Second Editorial decision:	10-Mar-2017
	Second Revision received:	16-Mar-2017
	Third Editorial decision:	20-Mar-2017
	Accepted:	30-Mar-2017

Handling Executive Committee member: Prof. Hans-Martin Jäck
Please note that the correspondence below does not include the standard editorial instructions regarding preparation and submission of revised manuscripts, only the scientific revisions requested and addressed.

First Editorial Decision

23-Jan-2017

Dear Dr. Schneider,

Manuscript ID eji.201746934 entitled "A mouse model of systemic lupus erythematosus responds better to soluble TACI than to soluble BAFFR after onset of autoimmunity, correlating with depletion of bone marrow plasma cells" which you submitted to the European Journal of Immunology has been reviewed. The comments of the Executive Editor are included at the bottom of this letter.

You should also pay close attention to the editorial comments included below. **In particular, please

edit your figure legends to follow Journal standards as outlined in the editorial comments. Failure to do this will result in delays in the re-review process.**

If the revision of the paper is expected to take more than three months, please inform the editorial office.

Once again, thank you for submitting your manuscript to European Journal of Immunology and we look forward to receiving your revision.

Yours sincerely,
Nadja Bakocevic

On behalf of
Prof. Hans-Martin Jäck

Dr. Nadja Bakocevic
Editorial Office
European Journal of Immunology
e-mail: ejied@wiley.com
www.eji-journal.eu

****

Executive Editor:

The findings reported by the authors are interesting. However, before we can consider the manuscript for an outside review, the authors must address the following points:

1. In Fig. 2, the analysis of plasma cells numbers is not plausible because the authors did not specially mention how they enumerated numbers of plasma cells. To solve this problem, the authors must include as supplements a description of the staining procedure, and most importantly, the original flows diagrams with their gating strategy.

2. The authors should go through all labels of x and y axes in their diagrams. For example, in Fig. 2B it is not clear whether they documented frequencies or absolute cell numbers. The latter one is absolutely necessary for analyzing the effect on a cell population. The authors should check legends and labeling of all other figures for clarity.

First Revision – authors' response
28-Jan-2017

Dear Executive Editor,

Thank you for taking a look at our manuscript. We are pleased that you find it interesting. Please find below a point by point answer to your comments:

Answer to comment 1 (Provide gating strategies):

We have added a Suppl Figure 2 with the gating strategies that we have used to identify CD138+ cells in the spleen and in the bone marrow, and B220+/CD19+ cells in the spleen. We apologize for not having provided this figure in the first place.

Answer to comment 2 (Clarify legends to x- and y-axes):

It was unclear to the executive Editor if the legend of the y-axis in Fig 2B (CD138+ PC in spleen [millions]) documented frequencies or absolute cell numbers. The answer is that this axis documents absolute cell numbers. We have changed the legend of the axis to "CD138+ PC [millions/spleen]" and changed the figure legend "Cell numbers were determined..." to "Absolute cell numbers were determined...". A similar case applied to Fig. 2D, where we have changed the y-axis from "CD138+ PC in BM [thousands]" to "CD138+ PC [thousands/2 femurs]" and the figure legend to "Same as panel B, but for absolute numbers of CD138+ cells in the bone marrow of 2 femurs ....".

We have added in the figure legends comments to mention whenever we measured absolute cell numbers.

Finally, we have edited the figure legends to follow Journal standards as recommended.

Changes made are highlighted in yellow in the manuscript and in supplemental file (we uploaded them as "Supporting information not for review"). Clean versions were uploaded as "Submission file" and "Supporting information for review".

Yours sincerely

Pascal Schneider

Second Editorial Decision

10-Mar-2017

Dear Dr. Schneider,

We are sorry for a slight delay in the peer review of your revised manuscript ID eji.201746934.R1 entitled "A mouse model of systemic lupus erythematosus responds better to soluble TACI than to soluble BAFFR after onset of autoimmunity, correlating with depletion of bone marrow plasma cells" to the European Journal of Immunology. Your manuscript has been re-reviewed and the comments of the referees are included at the bottom of this letter.

Although the referees have recommended publication, some revisions to your manuscript have been requested. Therefore, I invite you to respond to the comments of the referee 2 and revise your manuscript accordingly.

You should also pay close attention to the editorial comments included below. **In particular, please edit your figure legends to follow Journal standards as outlined in the editorial comments. Failure to do this will result in delays in the re-review process.**

If the revision of the paper is expected to take more than three months, please inform the editorial office. Revisions taking longer than six months may be assessed by new referees to ensure the relevance and timeliness of the data.

Once again, thank you for submitting your manuscript to European Journal of Immunology. We look forward to receiving your revision.

Yours sincerely,
Nadja Bakocevic

on behalf of
Prof. Hans-Martin Jäck

Dr. Nadja Bakocevic
Editorial Office
European Journal of Immunology
e-mail: ejied@wiley.com

www.eji-journal.eu

Get the latest articles delivered directly to your desktop ! Register now for the free Wiley Alerting Service at
http://onlinelibrary.wiley.com/myprofile/alertManager

****

Reviewer: 1

Comments to the Author
I have got the revised version of this paper for review.
This paper address a relevant issue, modulating the BAFF-APRIL system as a therapy for autoimmune diseases. Their results are clear. In this model a soluble receptor blocking BAFF only is less effective than a soluble receptor that blocks both BAFF+APRIL. This is not really surprising. Nevertheless, such data are informative, since anti-BAFF, but atacicept (blocking BAFF+APRIL) is not yet approved in human SLE.
The authors have adequately addressed the issues raized by the reviewers of the original submission.

Reviewer: 2

Comments to the Author
In general the study is very well performed and the experiments are clearly described and discussed including possible limitations of the study. The study is clearly of relevance and interest.

There are however a few minor points that should be addressed before publications.

Figure 1B: Legend is missing. I assume same legend as in Figure 1C but no mTACI-Fc shown in this panel and not included in the legend.
P5, line 55 and Figure 1D: How long after depletion was the FACS analysis performed?

In Figure 2E. ANA titres were assumed to stay constant if a mouse had to be euthanized before the conclusion of the experiment. Is that a reasonable assumption and leave out the points that were not actually measured from the graph 2E?

Figure 4A/B: Dual lines for statistical significance is confusing. No stats shown on the hTACI-Fc group?

Is there a possible explanation for the production of autoantibodies in the NZB/NZW F1 mice but not in the C57BL/6 mice and would that potentially be relevant for a clinical application of the human equivalent to Apry-1-1?

Second Revision – authors' response
16-Mar-2017

Point-by-point answer to the comments of reviewers.

Reviewer: 1

Comments to the Author
I have got the revised version of this paper for review.
This paper addresses a relevant issue, modulating the BAFF-APRIL system as a therapy for autoimmune diseases. Their results are clear. In this model a soluble receptor blocking BAFF only is less effective than a soluble receptor that blocks both BAFF+APRIL. This is not really surprising. Nevertheless, such data are informative, since anti-BAFF, but atacicept (blocking BAFF+APRIL) is not yet approved in human SLE.
The authors have adequately addressed the issues raised by the reviewers of the original submission.

Answer: We thank reviewer 1 for her/his positive comments.

Reviewer: 2

Comments to the Author
In general the study is very well performed and the experiments are clearly described and discussed including possible limitations of the study. The study is clearly of relevance and interest.

There are however a few minor points that should be addressed before publications.

Point 1:
Figure 1B: Legend is missing. I assume same legend as in Figure 1C but no mTACI-Fc shown in this panel and not included in the legend.

Answer: The reviewer is right. We have now identified symbols in panel 1B, including mTACI-Fc.

Point 2:
P5, line 55 and Figure 1D: How long after depletion was the FACS analysis performed?

Answer: Inhibitors were administered 3 times a week on a Monday/Wednesday/Friday schedule for 12 weeks. At the end of the experiment, mice were sacrificed two days after the last administration of inhibitors. In some cases, this time was reduced to 1 day (for some animal that had to be sacrificed before the end of the experiment). At those time points, BAFF inhibitory activity was still measurable in the circulation (for those inhibitors that inhibit BAFF) (Fig. 1E).

Point 3:
In Figure 2E. ANA titres were assumed to stay constant if a mouse had to be euthanized before the conclusion of the experiment. Is that a reasonable assumption and leave out the points that were not actually measured from the graph 2E?

Answer: The reviewer is right: our graph was not optimal. In its updated version, the last measure is shown at the actual time point of measurement and sacrifice is marked by termination of the line with a black circle.

Point 4:
Figure 4A/B: Dual lines for statistical significance is confusing. No stats shown on the hTACI-Fc group?

Answer: In a statistical comparison by one way ANOVA, a group very different from others obliterates differences between less dissimilar groups, even if real. We discuss in the manuscript the exceptional behavior of hTACI-Fc in vivo compared to other inhibitors tested (mTACI-Fc, mBAFFR-Fc and Apry1.1). When hTACI-Fc is included in the analysis, nothing else scores significant. However, differences are significant when hTACI-Fc is excluded from the analysis, i.e. when the control is compared only to inhibitors of comparable potency. Similarly, the weak APRIL-inhibitory activity of mTACI-Fc is significant when compared to controls (bottom line of statistical significance in panel B), but not when more potent inhibitors are included in the analysis. This is the reason why we showed dual lines for statistical analysis. We have added the following explanation in the legend: “For panels A and B, only hTACI-Fc is significantly different from all other groups by one way Anova ($p<0.001$). The line of statistical results that is shown in panel A compares controls to inhibitors of similar potency (i.e excluding the group of hTACI-Fc) by one way Anova. For panel B, inhibitors of medium activity (Apry-1-1, upper line of statistical results) and of weak activity (mTACI-Fc, lower line of statistical results) are independently compared to

controls by one way Anova”.

Point 5:
Is there a possible explanation for the production of autoantibodies in the NZB/NZW F1 mice but not in the C57BL/6 mice and would that potentially be relevant for a clinical application of the human equivalent to Apry-1-1?

Answer: The reviewer raises an interesting point. Apry-1-1 is a single chain human antibody fused to the Fc portion of mouse IgG2b. In the presence of adjuvant, this will certainly be immunogenic in non-autoimmune mice also. In NZB/NZW F1, B cells can recognize and neutralize Apry-1-1 in the absence of adjuvant because they are prone to activation. We think that a fully mouse anti-mAPRIL antibody should not be immunogenic, or much less, in NZB/NZW F1 mice, unless anti-idiotype responses would take place, which is possible. Whether a fully human anti-hAPRIL antibody in autoimmunity-prone humans will elicit an anti-drug response is not a trivial question. Maybe, may be not. Time will tell.

We thank reviewer 2 for her/his input.

Third Editorial Decision

20-Mar-2017

Dear Dr. Schneider,

It is a pleasure to provisionally accept your manuscript entitled "A mouse model of systemic lupus erythematosus responds better to soluble TACI than to soluble BAFFR, correlating with depletion of plasma cells" for publication in the European Journal of Immunology. For final acceptance, please follow the instructions below and return the requested items as soon as possible as we cannot process your manuscript further until all items listed below are dealt with.

Please note that EJI articles are now published online a few days after final acceptance (see Accepted Articles: http://onlinelibrary.wiley.com/journal/10.1002/(ISSN)1521-4141/accepted). The files used for the Accepted Articles are the final files and information supplied by you in Manuscript Central. You should therefore check that all the information (including author names) is correct as changes will NOT be permitted until the proofs stage.

We look forward to hearing from you and thank you for submitting your manuscript to the European Journal of Immunology.

European Journal of Immunology

Yours sincerely,
Nadja Bakocevic

on behalf of
Prof. Hans-Martin Jäck

Dr. Nadja Bakocevic
Editorial Office
European Journal of Immunology
e-mail: ejied@wiley.com
www.eji-journal.eu
